# NMR-Based Metabolomic Analysis of Cardiac Tissues Clarifies Molecular Mechanisms of CVB3-Induced Viral Myocarditis and Dilated Cardiomyopathy

**DOI:** 10.3390/molecules27186115

**Published:** 2022-09-19

**Authors:** Qing Kong, Jinping Gu, Ruohan Lu, Caihua Huang, Lili Chen, Weifeng Wu, Donghai Lin

**Affiliations:** 1 Department of Cardiology, The First Affiliated Hospital of Guangxi Medical University, Nanning 530021, China; 2 Key Laboratory for Chemical Biology of Fujian Province, MOE Key Laboratory of Spectrochemical Analysis & Instrumentation, College of Chemistry and Chemical Engineering, Xiamen University, Xiamen 361005, China; 3 Key Laboratory for Green Pharmaceutical Technologies and Related Equipment of Ministry of Education, College of Pharmaceutical Sciences, Zhejiang University of Technology, Hangzhou 310014, China; 4 Research and Communication Center of Exercise and Health, Xiamen University of Technology, Xiamen 361024, China

**Keywords:** viral myocarditis, dilated cardiomyopathy, metabolomics, NMR, cardiac tissue

## Abstract

Viral myocarditis (VMC), which is defined as inflammation of the myocardium with consequent myocardial injury, may develop chronic disease eventually leading to dilated cardiomyopathy (DCM). Molecular mechanisms underlying the progression from acute VMC (aVMC), to chronic VMC (cVMC) and finally to DCM, are still unclear. Here, we established mouse models of VMC and DCM with Coxsackievirus B3 infection and conducted NMR-based metabolomic analysis of aqueous metabolites extracted from cardiac tissues of three histologically classified groups including aVMC, cVMC and DCM. We showed that these three pathological groups were metabolically distinct from their normal counterparts and identified three impaired metabolic pathways shared by these pathological groups relative to normal controls, including nicotinate and nicotinamide metabolism; alanine, aspartate and glutamate metabolism; and D-glutamine and D-glutamate metabolism. We also identified two extra impaired metabolic pathways in the aVMC group, including glycine, serine and threonine metabolism; and taurine and hypotaurine metabolism Furthermore, we identified potential cardiac biomarkers for metabolically distinguishing these three pathological stages from normal controls. Our results indicate that the metabolomic analysis of cardiac tissues can provide valuable insights into the molecular mechanisms underlying the progression from acute VMC to DCM.

## 1. Introduction

Myocarditis is defined as inflammation of the myocardium with consequent myocardial injury. The leading cause of myocarditis is infection with enteroviruses, especially cardiotropic Coxsackievirus B3 (CVB3). CVB3-induced acute myocarditis (aVMC) can either cause sudden death or induce chronic myocarditis (cVMC), even leading to the development of DCM, a chronic condition in which the heart becomes weakened and enlarged. Patients with DCM have only a 5-year survival rate of 55% under current heart failure treatment [1]. Few efficient therapeutic measures have been developed to alleviate the progression from acute VMC to DCM owing to both unclear molecular mechanisms of the progression and lack of diagnostic biomarkers at an early pathological stage.

Clinical presentations of myocarditis are diverse, including nonspecific flu-like symptoms, palpitations, arrhythmias and acute cardiogenic shock. As a gold standard for suspected myocarditis in the last three decades, endomyocardial biopsy would provide false negative results (sampling error) if only small numbers of samples (<8–10) were available [2]. Actually, endomyocardial biopsy cannot be frequently conducted for safety reasons. Thus, noninvasive techniques are urgently needed for the diagnosis of myocarditis.

The heart is a metabolically active organ. As one of omics technologies, metabolome is downstream of the genome and closer to the organism phenotype. Recently, metabolomic analysis has emerged as a powerful tool for exploring changes in global and cardiac-specific metabolisms occurring across a spectrum of cardiovascular disease states [3]. Cardiovascular diseases are closely related to impaired cardiac metabolisms, including cardiac hypertrophy, heart failure, myocardial ischemia and infarction [4], aortic aneurysm and dissection [5]. Expectedly, alterations in metabolic phenotypes emerging before clinical symptoms might be identifiable.

In a previous study, we established mouse models of aVMC, cVMC and DCM by infecting BALB/c mice with CVB3 and showed that NMR-based serum metabolomic analysis can be applied to mechanistically understand the pathological progression from aVMC, cVMC to DCM [6]. As one of the powerful analysis techniques, NMR spectroscopy possesses remarkable advantages, such as noninvasive and non-destructive detection, high resolution and high experimental repeatability, as well as convenient quantification [7]. We showed that metabolic profiles of the three pathological stages were distinguished from their normal counterparts and identified significantly altered metabolic pathways in aVMC, cVMC and DCM stages relative to normal controls. Moreover, we identified potential serum biomarkers for discriminating these pathological stages from their normal controls, such as taurine, valine and acetate for the aVMC stage, glycerol, valine and leucine for the cVMC stage; and citrate, glycine and isoleucine for the DCM stage. These results are of benefit to improvements of clinical outcomes and exploitation of biomarkers for prognosis and diagnosis of CVB3-induced VMC and DCM.

Consistent with our previous study on sera derived from the mouse model of DCM [6], Ampong previously reported increased serum levels of acetate and lactate in DCM patients [8], Haas et al. observed the increased level of succinate [9], and Zhao et al. detected the increased level of creatine in DCM patients [10]. However, most of identified differential metabolites were different between DCM patients and DCM mice. These distinctions might be attributed to the fact that predisposing causes of DCM are unclear in clinic practice. Actually, human DCM potentially results from viral infection, bacterial infection, autoimmunity, or familial inheritance [1,2,3,4,5]. In the current study, we focused on the progression from aVMC to cVMC and DCM using the CVB3-infected mouse model with a known predisposing cause. Our results are expectedly complementary to those obtained from the previous studies on sera of DCM patients.

Due to the challenges in obtaining human heart samples, biofluids (sera, urine, etc.) samples are extensively used for most metabolomic studies on DCM patients. Biofluids usually reflect global pathological features of diseases [6,8,9,10]. Nevertheless, tissue metabolomic studies are preferred for studying specifically localized responses to stimuli and pathogenesis, providing explicit biochemical information about pathological mechanisms of diseases [11]. Compared with sera, cardiac tissues are more specifically and closely related to metabolic disorders in cardiovascular diseases [12,13,14]. Thus, metabolomic studies on cardiac tissues are crucial and necessary for mechanistically understanding physiological processes during the development of DCM.

Expectedly, metabolic profiling of cardiac tissues derived from aVMC, cVMC and DCM mice relative to their normal controls would provide a more accurate understanding of metabolic mechanisms underlying the progression from acute VMC to DCM. So far, few metabolomic analyses have been performed on cardiac tissues to address the metabolic mechanisms of VMC progressing into DCM in detail. Based on the established mouse models of aVMC, cVMC and DCM, we performed NMR-based metabolomic analyses of cardiac tissues, identified potential cardiac biomarkers and impaired metabolic pathways in the three pathological groups relative to normal counterparts. Our results shed light on the molecular mechanisms underlying the progression from acute VMC to DCM.

## 2. Results

### 2.1. Viral Myocarditis and Dilated Cardiomyopathy Induced by CVB3 in Mice

In total, 48 mice were used for metabolomic analysis, including 28 MODEL mice (aVMC, cVMC and DCM) and 20 CON mice (CON-w2, CON-w6 and CON-w24). The detailed histologic examination of the mouse models has been described in our previous study [6]. Regarding the MODEL mice, serious cardiac inflammation was observed and gradually reduced in the aVMC mice. Fibrosis was gradually promoted during the pathological progression with the highest level in the DCM mice. Moreover, only the DCM mice emerged with increased cavity dilation and decreased wall thickness of ventricles. These data indicated that the mouse models of aVMC (week 2), cVMC (week 6) and DCM (week 24) were successfully established by inducing with CVB3 infection. Furthermore, as described in our previous study [6], cardiac CVB3 mRNA levels and viral titers were not gradually increased by monthly CVB3 injection, indicating that metabolic disorders in the cVMC and DCM stages mostly resulted from the pathological progression rather than the monthly CVB3 injection. Concrete details were described in our previous study [6].

### 2.2. Metabolic Alterations in CVB3-Infected Groups Compared with Normal Controls

**Figure 1:**
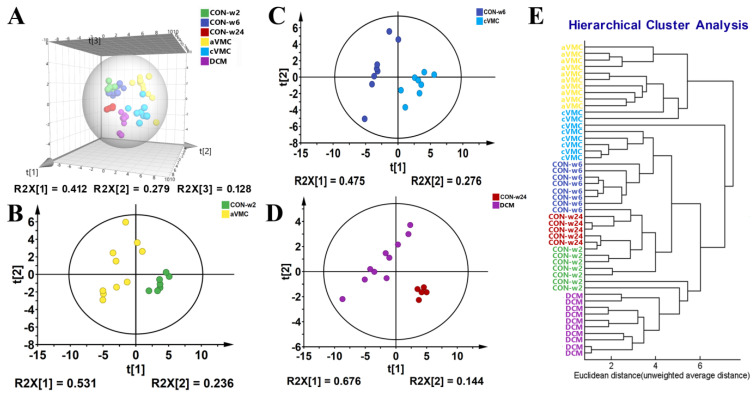
Multivariate statistical analyses for 1D ^1^H−NMR spectra recorded on the six groups of mouse cardiac tissues (CON−w2, CON-w6, CON−w24, aVMC, cVMC and DCM): (**A**) PCA scores plot for the six groups; (**B**–**D**) PCA scores plots for aVMC and CON−w2 (**B**); cVMC and CON−w6 (**C**); DCM and CON−w24 (**D**); (**E**) HCA clustering tree for the six groups. Each point represents a sample derived from an individual mouse.

A total of 25 metabolites were identified from the typical 1D ^1^H-NMR spectrum of mouse cardiac tissue (Table S1, Figures S1 and S2). To obtain overall metabolic information and examine metabolic profiles of the six groups of cardiac tissues, we performed unsupervised HCA and PCA analyses on the NMR data sets of three CVB3-infected groups (aVMC, cVMC and DCM) and their normal counterparts (CON-w2, CON-w6 and CON-w24). The three control groups displayed indistinguishable metabolic profiles (Figure 1A,E). However, the aVMC, cVMC and DCM groups exhibited distinctly different metabolic profiles from the CON-w2, CON-w6 and CON-w24 groups, respectively (Figure 1B–D).

To maximize metabolic distinctions between the CVB3-infected groups and normal controls, we constructed six pairwise OPLS-DA models with tp1 based on the NMR data sets of the cardiac tissues. The OPLS-DA score plots showed distinct metabolic separations between the pathologic groups and normal controls (Figure S3A–C). Furthermore, we performed response permutation tests (RPTs) with 200 cycles to validate the robustness of the OPLS-DA models, which showed that these models were not overfitting (Figure S4). The obtained R2, Q2 and *p* values were 0.921, 0.878 and 0.005 for aVMC vs. CON-w2, 0.842, 0.733 and 0.005 for cVMC vs. CON-w6 and0.792, 0.724 and 0.005 for DCM vs. CON-W24. Totally, the OPLS-DA loading plots identified 13, 17 and 8 significant metabolites for the pairwise comparisons of aVMC vs. CON-w2, cVMC vs. CON-w6 and DCM vs. CON-w24, respectively (Figure S3D–F).

We normalized NMR integrals of metabolites by the tissue weight and the integral of TSP to obtain relative levels of metabolites. Then, we conducted Student’s t-test to quantitatively compare relative levels of cardiac metabolites in these three pathologic groups relative to their normal controls (Table 1). Totally, 13, 13 and 15 differential metabolites were identified for the pairwise comparisons of aVMC vs. CON-w2, cVMC vs. CON-w6 and DCM vs. CON-w24, respectively. Then, 12, 13 and 8 characteristic metabolites were identified for the pair-wise comparisons between these three pathologic groups and their normal counterparts (Table 1).

### 2.3. Amino Acid Metabolism

Relative to normal controls, leucine, isoleucine and valine (branch chain amino acids, BCAAs) showed relative stable levels in the aVMC group and profoundly upregulated levels in the cVMC group. Leucine was decreased, but isoleucine and valine were not significantly altered in the DCM group. Threonine was decreased in aVMC and DCM groups but not observably changed in the cVMC group. Glycine was remarkably decreased in the three pathologic groups relative to normal controls with the most significantly change in the aVMC group. Lysine was markedly increased in the cVMC group but remained relative stable in the cVMC and DCM groups. Alanine was obviously decreased in the aVMC and DCM groups but basically was not changed in the cVMC group. Taurine was decreased in the aVMC group but not observably fluctuated in other groups. Glutamine was not observably altered in these three pathologic groups. Both glutamate and aspartate were obviously decreased in the aVMC and DCM groups without a detectable change in the cVMC group.

### 2.4. Carbohydrate Metabolism

Compared with normal controls, the cVMC and DCM groups exhibited downregulated levels of glucose, while the aVMC group did not show significantly altered level of glucose. Acting as two important metabolites of glycolysis, lactate and succinate were profoundly decreased in the aVMC and DCM groups without observable changes in the cVMC group. Moreover, creatine was remarkably decreased in the aVMC group but kept almost unchanged levels in the cVMC and DCM groups. Furthermore, dimethylamine and formate were significantly decreased, but acetate basically was not changed in all the three groups. In addition, fumarate was distinctly increased in the cVMC group but was not obviously changed in the aVMC and DCM groups.

### 2.5. Lipid Metabolism

As a crucial metabolite in lipid metabolism, 3-hydroxybutyrate (3-HB) was significantly increased in the cVMC group, but was almost unchanged in the aVMC and DCM groups.

### 2.6. Choline Phosphorylation Metabolism

After CVB3-infection, the choline phosphorylation metabolism of cardiac tissues became disordered. Relative to normal controls, glycerophosphotidylcholine (GPC) was remarkably decreased in the DCM group, but not observably changed in the aVMC and cVMC groups. Additionally, choline was upregulated in the cVMC and DCM groups without detectable change in the aVMC group.

### 2.7. Others

As well known, NAD^+^ and AMP are involved in energy metabolism. Compared with normal controls, the three pathological groups showed profoundly decreased levels of NAD^+^ and AMP and relative stable levels of myoinositol.

### 2.8. Potential Biomarkers in the Progression from Acute VMC to DCM

**Figure 2:**
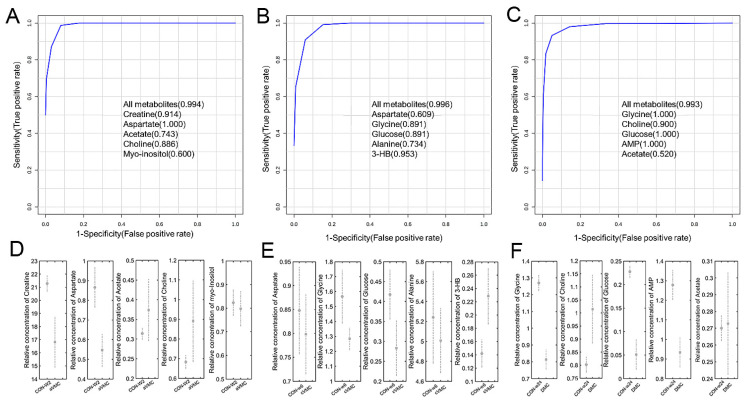
Multivariate ROC analyses of cardiac tissues for identifying potential biomarkers in the three pathological stages and comparisons of relative levels of potential identified biomarkers: (**A**–**C**) multivariate ROC analyses of aVMC vs. CON-w2 (**A**); cVMC vs. CON-w6 (**B**); DCM vs. CON-w24 (**C**); (**D**–**F**) comparisons of relative levels of potential biomarkers for aVMC vs. CON-w2 (**D**); cVMC vs. CON-w6 (**E**); DMC vs. CON-w24 (**F**).

Multivariable ROC analyses were performed on five metabolites to identify potential biomarkers in mouse heart tissues (Figure 2). Metabolites were ranked by the selection frequencies (Figure S5), and potential biomarkers were identified by two criteria of the selection frequency >0.4% and AUC >0.7. The aVMC stage showed large AUC values with either only one of the following metabolites or their combination: 0.914 for creatine; 1.000 for aspartate; 0.743 for acetate; and 0.994 for these five metabolites. The cVMC stage also displayed large AUC values: 0.891 for glycine; 0.891 for glucose; 0.734 for alanine; and 0.996 for these five metabolites. The DCM stage exhibited large AUC values: 1.000 for glycine; 0.900 for choline; 1.000 for glucose; 1.000 for AMP; and 0.993 for these five metabolites.

### 2.9. Significantly Altered Metabolic Pathways in the Progression from Acute VMC to DCM

**Figure 3:**
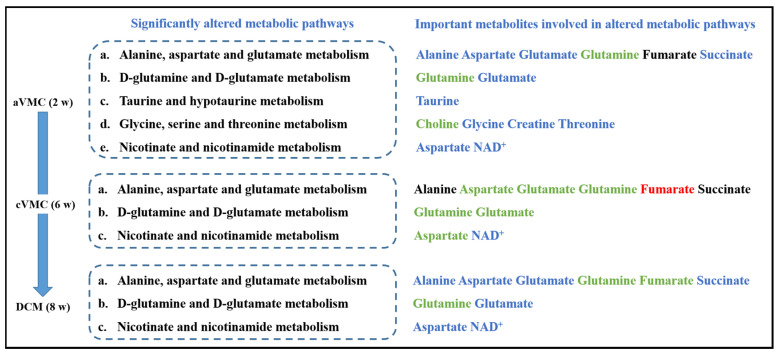
Schematic representation of significantly altered metabolic pathways associated with the three pathological stages compared with normal stages. Red, blue and black colors represent significantly increased, decreased and substantially unchanged metabolites; green colors denote the metabolites with the changing trends without statistically significance.

**Figure 4:**
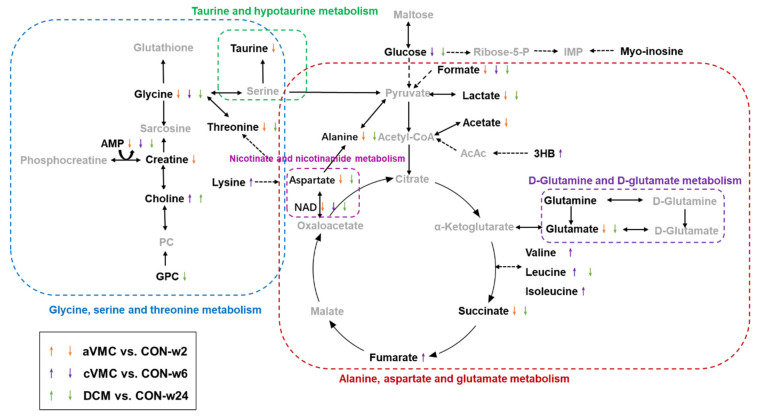
Overview of significantly impaired metabolic pathways involved in the three pathological stages compared with normal controls. The up/down arrows indicate significantly increased/decreased metabolites; dashed/solid arrows represent multi-step/single-step reactions.

We screened out significantly altered metabolic pathways in these three pathological stages relative to normal counterparts based on cardiac levels of all assigned metabolites using two criteria of PIV > 0.2 and *p* < 0.05 (Figure S6). Significantly, these pathological stages shared three impaired metabolic pathways, including nicotinate and nicotinamide metabolism; alanine, aspartate and glutamate metabolism; and D-glutamine and D-glutamate metabolism. Furthermore, the aVMC stage showed two extra impaired metabolic pathways including glycine, serine and threonine metabolism and taurine and hypotaurine metabolism(Figure 3). To visualize changes in levels of characteristic metabolites during the pathological progression from aVMC to DCM, we projected these metabolites onto a metabolic map based on the Kyoto Encyclopedia of Genes and Genomes (KEGG) database (Figure 4).

## 3. Discussion

CVB3 infection is greatly responsible for the development of myocarditis which primarily covers three pathological stages: acute VMC, chronic VMC and DCM [15]. To comprehensively understand the molecular mechanisms underlying the progression from acute VMC to DCM, we established mouse models of VMC and DCM with CVB3 infection and performed NMR-based metabolomic analysis of aqueous metabolites extracted from cardiac tissues of the aVMC, cVMC and DCM mice [6]. We showed that metabolic profiles of these three pathological groups were clearly distinguished from their normal counterparts and identified significantly altered metabolic pathways in these pathological groups relative to normal controls. Significantly, the present metabolic profiling of mouse cardiac tissues showed similar and dissimilar results to those obtained from our previous metabolic profiling of mouse sera. Levels of some significant metabolites showed consistent/opposite trends between cardiac tissues and sera (Table S2). For example, consistent with sera, cardiac tissues showed decreased levels of glycine and taurine in the aVMC stage, increased levels of leucine, isoleucine, valine and lysine in the cVMC stage and decreased levels of leucine and GPC in the DCM stage. These results suggest that the changing trends of serum levels of some significant metabolites in circulation could reflect those of their corresponding cardiac levels to an extent. In contrast, levels of some other significant metabolites exhibited the opposite changing trends, such as increased serum levels of glycine, glucose, lactate and succinate but decreased cardiac levels in the DCM stage.

Notably, as one of the biomarkers identified from both cardiac tissue and serum samples, acetate is the unique metabolite to the aVMC stage. Previous studies have demonstrated that acetate can restrain LPS-induced TNF-αsecretion from mice and human mononuclear cells [16] and also inhibit the expressions of IL-6, IL-1β and TNF-α to exert anti-inflammatory effects [17,18,19]. Moreover, it was reported that acetate can be converted into acetyl-CoA, which enters the tricarboxylic acid cycle to produce energy [20]. Thus, acetate is capable of inhibiting cardiac inflammation and improving energy production after early infection with CVB3. Furthermore, glycine was a biomarker of the DCM stage shared by cardiac tissue and serum samples. Previous studies indicated that elevated levels of glycine provide protection from cardiovascular events [21], supporting the result that glycine, serine and threonine metabolism was identified as the significant metabolic pathway in DCM patients [12,13,14]. The protective role of glycine in the heart might be related to its capability of decreasing levels of TNF-α, IL-1β, IL-6 and increasing the level of IL-10, which is indicative of the anti-inflammatory effect of glycine in humans [22,23]. Additionally, it was previously reported that glycine can increase both the expression of collagen and the content of collagen I (the predominance type of collagen) [24], implying a role of glycine in promoting cardiac remodeling. Further studies should be conducted to exploit and confirm the roles these important metabolites play in the pathological progression of VMC into DCM based on the mouse model of DCM, which are shared by cardiac tissue and serum samples. Such studies would be of benefit to exploring the potentials of those biomarkers identified from serum samples for diagnosing cardiomyopathies.

### 3.1. Alanine, Aspartate and Glutamate Metabolism Is Disordered during the Progression from Acute VMC to DCM

In this study, we found that alanine, aspartate and glutamate were remarkably decreased in aVMC and DCM mice. A previous study showed that the pretreatment using either aspartate or glutamate in rats with myocardial infarction can significantly decrease the activities of cardiac marker enzymes but increase the activities of antioxidant enzymes, preventing cardiac damage through reducing oxidative stress [25]. Expectedly, alanine, aspartate, and glutamate metabolism might play important roles in macrophage survival and cytokine-mediated inflammation [26], potentially alleviating the inflammation in ulcerative colitis [27]. Thus, these obviously decreased amino acids potentially contributed to the serious cardiac inflammation in aVMC mice and fibrosis in DCM mice.

### 3.2. Nicotinate and Nicotinamide Metabolism Is Impaired during the Progression from Acute VMC to DCM

During the progression from acute VMC to DCM, nicotinate and nicotinamide metabolism is impaired, which is identified from metabolic pathway analyses. The downregulated levels of nicotinamide adenine dinucleotide (NAD^+^) and aspartate were only detected in VMC and DCM mice from cardiac samples, indicating impaired nicotinate and nicotinamide metabolism in CVB3-infected mice. We found that cardiac NAD^+^ was decreased during the progression from aVMC to DCM. As reported by Nicolas Diguet et al., both the early stage of murine DCM and human failing heart biopsies showed that the levels of NAD^+^ were decreased by about 30% relative to normal controls, and the supplementation of NAD precursors in food could attenuate those pathological conditions [28]. As the major hydride transfer coenzyme in fuel oxidation and mitochondrial ATP generation, NAD^+^ plays crucial roles in anabolic pathways and ROS detoxification [29,30,31]. Whether dietary NAD^+^ precursors are beneficial to treatments of VMC and DCM, especially to improvement of energy supplies for cardiac tissues, remains to be determined in future. On the other hand, amino acids could be converted into TCA intermediates and enter a TCA cycle to generate ATP. In this study, the MODEL mice displayed a disordered TCA cycle compared with their normal counterparts. Consistent with the data from DCM patients [22], circulatory succinate was increased in DCM mice. Cardiac succinate distinctly decreased in aVMC and DCM mice, indicating lower energy supplements in aVMC and DCM stages. Note that succinate serves as one of the TCA intermediates.

### 3.3. D-Glutamine and D-Glutamate Metabolism Is Altered during the Progression from Acute VMC to DCM

The present metabolic pathway analysis of cardiac tissues showed that D-glutamine and D-glutamate metabolism is significantly altered during the progression from acute VMC to DCM, which is different from our previous metabolic pathway analysis of sera [6]. Glutamine usually acts as one of the most abundant amino acids secreted by cardiac tissues [32]. Both glutamine and glutamate displayed fluctuating levels during the progression from aVMC to DCM, suggesting that the myocardium preferentially utilizes glutamate to maintain glutamine homeostasis.

Consistent with the result of our previous study performed on sera, leucine, isoleucine and valine were profoundly increased in cardiac tissues of cVMC mice, and leucine was significantly decreased in the DCM stage [6]. As increased branched-chain amino acids (BCAAs) can promote cardiac hypertrophy and muscle lipid accumulation contributing to insulin resistance, BCAAs are thereby considered as biomarkers for diagnosing cardiometabolic diseases [33]. Haipeng Sun et al. [34] showed that BCAA catabolic defect could promote heart failure associated with reduced oxidative stress and metabolic disturbance in response to mechanical overload. However, it is unclear whether the increased levels of BCAAs in the cVMC stage are related to critical metabolic reprogramming for the pathogenesis of DCM.

As the most important ketone, 3-HB is associated with promoted myocardial energy expenditure [35]; 3-HB dramatically increased in cardiac tissues of cVMC mice and significantly increased in sera of DCM mice [6]. On the other hand, consistent with the result of metabolic profiling on sera [6], cardiac lysine increased dramatically in cVMC mice. Lysine acetylation is usually related to myocardial ischaemia–reperfusion injury, cardiac hypertrophy, fibrosis, apoptosis and inflammation, showing the beneficial effect of abating cardiac and vessel injury [36,37]^.^ Further study is required to address the functions of lysine in the pathology of cVMC. In addition, both aVMC and DCM mice displayed significantly downregulated levels of lactate. As a glycolytic product, lactate is associated with the delivery of oxidative and gluconeogenic substrates, as well as cell signaling in cardiomyocytes with cardiac fibroblasts [38,39]. The role of lactate in cell signaling of cardiac tissue should be exploited in the future.

### 3.4. Glycine, Serine and Threonine Metabolism Is Disturbed in the Acute VMC Stage

Several studies have demonstrated that glycine, serine and threonine metabolism is impaired in cardiovascular diseases such as hypertrophic cardiomyopathy [40] and coronary artery disease [35]. This metabolic pathway supplies important precursors of energy metabolism for the citrate cycle [40] and reduces myocardial inflammation by activating glycine receptors, inhibiting collagen production in cardiac fibroblasts [41]. Our results showed that the levels of glycine and threonine were profoundly decreased in sera and cardiac tissues of aVMC mice, suggesting that supplements of these amino acids in aVMC mice might potentially inhibit cardiac inflammation and fibrosis.

The levels of GPC were decreased in sera and cardiac tissues of DCM mice. GPC is readily degraded to produce choline, potentially attenuating cardiac dysfunction through regulating expressions of the proteins involved in ketone body and fatty acid metabolism [42]. Furthermore, it has been demonstrated that decreasing the level of GPC is beneficial to protecting cardiac myocytes from cell death [43]. This study identified impaired choline phosphorylation metabolism in cardiac tissues, as indicated by the increased levels of choline in cVMC and DCM mice, and the remarkably decreased level of GPC in DCM mice, which might alleviate myocardial remodeling.

It is known that the mammalian cardiac tissues switch the main metabolic substrate from glucose to fatty acids shortly after birth and promoted glucose metabolism favors cardiac regeneration in neonatal mouse heart [44]. Our results showed that the levels of glucose were remarkably declined in cVMC and DCM mice, indicating that the metabolic switch might lead to the loss of regenerative capacity in cardiac tissues.

### 3.5. Taurine and Hypotaurine Metabolism Is Disordered in the aVMC Stage

Notably, both metabolic pathway analyses of sera and cardiac tissues show that taurine and hypotaurine metabolism is disordered in the aVMC stage [6], indicating the significant contribution of this metabolic pathway to the aVMC stage. Taurine participates in many physiological processes, including cellular oxidative stress and cell signaling. In this study, taurine decreased in aVMC mice but was basically unchanged in cVMC and DCM mice, implying that taurine and hypotaurine metabolism was disordered in the aVMC stage only. One possible explanation is that taurine and hypotaurine metabolism primarily contributes to oxidative stress and cell signaling in aVMC. Previous studies have demonstrated that the decreased level of creatine is related to the impaired myocardial energy metabolism [45]. Therefore, the observation that creatine was decreased in aVMC mice might be indicative of impaired myocardial energy metabolism. Note that creatine was identified as a potential biomarker in the aVMC stage (AUC = 0.914).

Amino acids serve as building blocks for protein synthesis and substrates for providing energy in cardiac tissues. Furthermore, some amino acid derivatives such as taurine, creatine, acetate, succinate, dimethylamine and formate are critical to bioenergenesis in the heart [46]. As the substrate for ATP synthesis [47], AMP was decreased in aVMC, cVMC and DCM mice relative to their normal controls, consistent with the changing trends of levels of these amino acid derivatives. These results imply the lack of energy supplement in cardiac tissues of CVB3-infected mice. Furthermore, similarly to the myocarditis stage, significantly increased levels of cardiac BCAA metabolites (leucine, isoleucine, valine) and fumarate were detected in myocarditis caused by trypanosoma cruzi infection (21 days post-infection) [48]. Regarding metabolomic profiling of the DCM stage, similar changing tendencies of metabolite levels were detected in other animal models or patients, including significantly decreased levels of cardiac aspartate [49] and distinctly increased levels of plasma glutamine [50], creatine [51], acetate and glucose [52], lactate and succinate [9].

Taken together, our results suggest that the biochemical mechanisms involved in the progression from aVMC to cVMC and DCM might be attributed to the lack of energy supplement, the fluctuation of anti-inflammatory metabolites, metabolic remodelling in the TCA cycle and the promotion of collagen production. Note that this suggestion is only based on a smaller scale of samples derived from mice. A larger scale of samples needs to be used to confirm the effectiveness of this suggestion. Additionally, our results showed that the three pathological stages were metabolically clearly distinguished from their normal counterparts, indicative of severe metabolic impairments during the progression from acute VMC to DCM. Given crucial roles of regulatory metabolic enzymes in the impaired metabolic pathways, both expression levels and activities should be measured. Such studies should be carried out in the future.

## 4. Materials and Methods

### 4.1. Experimental Animal and Ethical Approval

As described in our previous study [6], inbred male BALB/c mice (4–5 weeks of age) were inflected with either CVB3 (the MODEL mice) or PBS (the CON mice) following the protocol described in published studies [53,54]. The MODEL mice were divided into three groups, which were separately sacrificed at three time points: week 2 for aVMC (n = 10), week 6 for cVMC (n = 8) and week 24 for DCM (n = 10). Their corresponding CON mice were also separately sacrificed at three time points: week 2 for CON-w2 (n = 7), week 6 for CON-w6 (n = 8) and week 24 for CON-w24 (n = 5). Cardiac tissues of the mice were removed aseptically as fresh specimens for the following experiments. The experimental animal protocol (2021 KY-E-315) was approved by Ethical Review Committee of First Affiliated Hospital of Guangxi Medical University, China. All animal experiments were performed in accordance with relevant guidelines and regulations.

### 4.2. Histological Analysis

Heart samples were embedded in paraffin wax, and 4-µm to 5-µm histological sections of the paraffin-embedded tissues were stained with hematoxylin-eosin to assess myocardium inflammation. Masson’s trichrome staining was used to detect collagen deposition. Sections were scored by at least two individuals blinded to analyzed subjects [55,56].

### 4.3. Sample Preparation and NMR Analysis

Following the extraction protocol [57], cardiac tissues were thawed on ice prior to NMR experiments, and about 100 mg of each sample was cut out and then transferred into Eppendorf tubes. CH_3_OH was added into the cardiac samples (4 mL/g) with homogenization for 5 min; then H_2_O was added (2.854 mL/g) with homogenization for 2 min. Finally, CHCL_3_ was added (4 mL/g) with homogenization for another 2 min. After vortexing for 2 min, these tubes were centrifuged at 12,000 rpm for 15 min at 4 °C. After centrifugation, the water phase was taken, and CH_3_OH were removed completely by a nitrogen blowing concentrator. Then, these samples were freeze-dried to obtain water-soluble extracts in powder form, and aqueous extracts were reconstituted in 550 μL of NMR buffer (50 mM PBS, 1 mM TSP, pH 7.4) containing 10% D_2_O in Eppendorf tubes. These tubes were centrifuged at 3000× *g* for 3 min at 4 °C. Thereafter, these cardiac samples were transferred into 5-mm NMR tubes, which were centrifuged at 1000× *g* for 5 min.

All NMR experiments were conducted on a Bruker AVANCE III 600 MHz spectrometer at 298 K; 1D ^1^H-NMR spectra were recorded on cardiac samples using the pulse sequence NOESYGPPR1D [RD-G1-90°-t1-90°-τ_m_-G2-90°-ACQ] with water suppression during the relaxation delay and mixing time. A fixed total spin–spin relaxation delay of 80 ms was used to attenuate broad NMR signals of slowly tumbling macromolecules with short T_2_ relaxation times and simultaneously to retain signals of metabolites with low molecular weights. Experimental parameters were shown as follows: spectral width = 12 KHz; number of time domain data points (TD) = 64 K; relaxation delay (RD) = 4 s; acquisition time (ACQ) = 2.73 s; number of scans (NS) = 32. These NMR spectra were multiplied by an exponential function with a line-broadening factor of 0.3 Hz prior to Fourier transformation, automatically phased and corrected for baseline distortion carefully and referenced to the methyl group of 3-(trimethylsilyl) propionate-2,2,3,3-d4 (TSP, δ 0.00).

The water resonance regions of δ 5.2–4.65 were removed from the spectra. The spectral peaks were aligned with the Icoshift program. Then, the regions of δ 9.5–0.8 were binned by 0.001 ppm for statistical analysis. Peak integrals for each NMR spectrum were normalized by the TSP spectral integral and the weight of the tissue slice. The relative integral of each metabolite was calculated based on both the relative integral of singlet or non-overlapped peaks in each NMR spectrum and the proton number of the chemical group contained in the metabolite molecule, which was used to present the relative level of the metabolite. Resonances of aqueous metabolites derived from mouse cardiac tissues were assigned by a combination of Chenomx NMR Suite (Version 8.3, Chenomx Inc., Edmonton, AB, Canada), Human Metabolome Data Base (HMDB, http://www.hmdb.ca/; accessed on15 July 2022) and relevant literature. The resonance assignments were confirmed by using 2D ^1^H-^13^C HSQC spectra.

### 4.4. Multivariate Statistical Analysis

The NMR data were preprocessed prior to the multivariate statistical analysis by the MestRoNova 9.0 software (Mestrelab Research S.L., La Coruna, Spain). To compensate for differences in heart tissue sizes, the spectral integrals were normalized by the weight of the tissue sample. Before multivariate statistical analysis, the data were scaled by using Pareto scaling in the SIMCA 14.0 software (Umetrics AB, Umea, Sweden). The unsupervised principal component analysis (PCA) was conducted to reveal grouping trends of samples, highlight outliers, and show clustering for observations using the SIMCA 14.0 software (Umetrics AB, Umea, Sweden). Then, the supervised orthogonal signal correction partial least squares discriminant analysis (OPLS-DA) was applied to maximally discriminate metabolic profiles between the groups of samples, and the response permutation test (RPT) with 200 cycles was used to evaluate the robustness of the OPLS-DA model. In addition, hierarchical clustering analysis (HCA) was performed on the binned spectral data to confirm the grouping trends revealed by PCA. In HCA, each sample was first treated as a separate cluster, and then the algorithm combined them until all the samples belonged to a cluster.

### 4.5. Quantitative Comparison of Metabolite Levels and Identification of Characteristic Metabolites

Before carrying out statistical analysis, we performed the KS test for checking statistical distributions of the relative levels of metabolites, which showed that the relative levels of metabolites basically followed normal distributions. The significance of variables (VIP) in the projection was calculated from the established OPLS-DA model, and metabolites with VIP > 1 were identified to be significant metabolites. To quantitatively analyze the levels of cardiac metabolites in each group, Student’s *t*-test was performed on relative levels of the metabolites. Metabolites with *p* < 0.05 were identified to be differential metabolites. Finally, metabolites with VIP > 1 and *p* < 0.05 were identified to be characteristic metabolites.

### 4.6. Metabolic Pathway Analysis

Metabolic pathway analysis was performed by using the MetaboAnalyst 5.0 website based on relative levels of cardiac metabolites. Metabolic pathway analysis is a good integration of metabolite aggregation enrichment analysis (MESA) and pathway topology analysis. As it is known, a metabolic pattern is closely related to the levels of metabolites acting as nodes of metabolic pathways in a metabolic network. Perturbations in metabolisms would change levels of key metabolites in relevant metabolic pathways. We calculated the pathway impact value (PIV) with the centrality algorithm of path topology and identified significantly altered metabolic pathways (important pathways) using two criteria of PIV > 0.2 and *p* < 0.05. The KEGG website was used to map the important pathways with changed metabolite levels into the metabolic network.

### 4.7. Multivariate Receiver Operating Characteristic Curve Analysis

Multivariate receiver operating characteristic (ROC) curve analyses were conducted to explore potential biomarkers using a logistic regression algorithm. We randomly selected 66.7% of cardiac samples for the ROC analyses of aVMC, cVMC and DCM mice compared with normal counterparts for screening potential biomarkers. Multivariate ROC curves were constructed by using the biomarker analysis module of MetaboAnalyst 5.0. Furthermore, multivariate ROC analyses were performed on the remaining 33.3% of the cardiac samples to confirm the validities of the identified potential biomarkers for predicting these pathological stages. The ROC curve area values (AUCs) were used to evaluate the predictive performances of the biomarker models. For each biomarker model with AUC > 0.7, metabolites with frequencies of being selected during cross validation (selection frequency) >0.4% were identified to be potential biomarkers for the diagnosis of a given pathological condition.

## 5. Conclusions

We established the mouse models of aVMC, cVMC and DCM, and performed NMR-based metabolomic analyses of cardiac tissues. We showed that these three pathological groups were metabolically distinct from their normal controls and identified three impaired metabolic pathways shared by these pathological stages, including nicotinate and nicotinamide metabolism; alanine, aspartate and glutamate metabolism and D-glutamine and D-glutamate metabolism. We also identified two extra impaired metabolic pathways in the aVMC stage, including glycine, serine and threonine metabolism and taurine and hypotaurine metabolism. In addition, we identified potential cardiac biomarkers for metabolically distinguishing these three pathological stages from normal counterparts. This study may be of benefit to a mechanistic understanding of the progression from acute VMC to DCM.

## Figures and Tables

**Table 1:** Relative levels of metabolites calculated from 1D ^1^H-NMR spectra of cardiac tissues derived from the six groups of mice.

	CON-w2	CON-w6	CON-w24	aVMC	cVMC	DCM
**Amino acid metabolism**						
leucine	0.693 ± 0.036	0.651 ± 0.064	0.616 ± 0.006	0.666 ± 0.110	0.823 ± 0.077↑ **	0.552 ± 0.057 *
isoleucine	0.132 ± 0.013	0.120 ± 0.021	0.124 ± 0.005	0.140 ± 0.024	0.195 ± 0.026↑ ***	0.112 ± 0.015
valine	0.253 ± 0.019	0.238 ± 0.030	0.246 ± 0.007	0.226 ± 0.043	0.319 ± 0.032↑ **	0.218 ± 0.025
threonine	1.928 ± 0.090	1.831 ± 0.118	1.918 ± 0.023	1.468 ± 0.146↓ ***	1.848 ± 0.056	1.667 ± 0.148 **
glycine	1.615 ± 0.086	1.567 ± 0.199	1.270 ± 0.042	1.378 ± 0.189 *	1.284 ± 0.084↓ *	0.813 ± 0.074↓ ***
lysine	0.851 ± 0.040	0.793 ± 0.086	0.813 ± 0.039	0.842 ± 0.167	1.089 ± 0.110↑ ***	0.747 ± 0.095
alanine	5.654 ± 0.122	5.242 ± 0.511	4.815 ± 0.260	3.911 ± 0.510↓ ***	5.008 ± 0.358	3.892 ± 0.415↓ **
taurine	38.357 ± 1.702	37.347 ± 3.062	38.409 ± 0.900	30.821 ± 3.664↓ ***	39.134 ± 2.041	35.727 ± 3.223
glutamine	7.763 ± 0.280	7.393 ± 0.614	7.944 ± 0.196	6.620 ± 1.081	8.164 ± 0.666	7.427 ± 0.680
glutamate	3.669 ± 0.193	3.288 ± 0.278	3.417 ± 0.127	2.768 ± 0.433↓ ***	2.985 ± 0.121	2.933 ± 0.330 *
aspartate	0.866 ± 0.106	0.848 ± 0.101	0.849 ± 0.052	0.547 ± 0.106↓ ***	0.798 ± 0.092	0.625 ± 0.094↓ ***
**Carbohydrate metabolism**						
creatine	21.283 ± 0.634	21.633 ± 1.790	23.039 ± 0.763	16.821 ± 2.414↓ **	22.511 ± 1.253	21.865 ± 2.261
acetate	0.314 ± 0.016	0.288 ± 0.033	0.270 ± 0.007	0.374 ± 0.097	0.323 ± 0.025	0.273 ± 0.037
glucose	0.360 ± 0.094	0.417 ± 0.069	0.228 ± 0.012	0.381 ± 0.095	0.283 ± 0.076↓ *	0.051 ± 0.040↓ ***
lactate	47.617 ± 1.393	43.705 ± 4.052	41.368 ± 3.622	36.914 ± 4.610↓ ***	44.133 ± 1.694	33.388 ± 3.968 *
succinate	3.761 ± 0.244	3.252 ± 0.481	3.520 ± 0.139	2.015 ± 0.330↓ ***	2.968 ± 0.316	2.969 ± 0.147 ***
dimethylamine	0.241 ± 0.033	0.229 ± 0.022	0.236 ± 0.014	0.138 ± 0.042↓ ***	0.125 ± 0.046↓ **	0.177 ± 0.024↓ ***
formate	0.164 ± 0.020	0.136 ± 0.015	0.186 ± 0.010	0.054 ± 0.018↓ ***	0.075 ± 0.023↓ ***	0.151 ± 0.011↓ **
fumarate	0.072 ± 0.011	0.055 ± 0.008	0.071 ± 0.010	0.077 ± 0.013	0.097 ± 0.016↑ ***	0.067 ± 0.012
**Lipid metabolism**						
3-HB	0.236 ± 0.051	0.142 ± 0.023	0.184 ± 0.027	0.182 ± 0.034	0.229 ± 0.047↑ **	0.164 ± 0.030
**Choline phosphorylation metabolism**						
GPC	5.585 ± 0.282	5.803 ± 0.463	5.792 ± 0.174	5.030 ± 0.576	6.241 ± 0.506	4.983 ± 0.494 **
Choline	0.683 ± 0.036	0.765 ± 0.119	0.802 ± 0.026	0.892 ± 0.256	1.089 ± 0.185↑*	1.015 ± 0.165↑ *
**Others**						
myoinositol	0.823 ± 0.057	0.798 ± 0.062	0.796 ± 0.014	0.789 ± 0.092	0.875 ± 0.058	0.722 ± 0.077
NAD	0.175 ± 0.021	0.152 ± 0.014	0.201 ± 0.009	0.078 ± 0.015↓ ***	0.093 ± 0.024↓ ***	0.166 ± 0.013 ***
AMP	1.428 ± 0.114	1.058 ± 0.071	1.278 ± 0.067	0.683 ± 0.116↓ ***	0.760 ± 0.142↓ **	0.933 ± 0.095↓ ***

**Note:** Stars ***, **, * mean that the changes of relative levels of metabolites in the MODEL groups are highly significant (*p* < 0.001), very significant (*p* < 0.01) and significant (*p* < 0.05) compared with their normal control groups (aVMC vs. CON-w2, cVMC vs. CON-w6 and DCM vs. CON-w24). Arrows ↑/↓ represent characteristic metabolites for comparisons of the MODEL groups with the CON groups, which were determined by a combination of significant metabolites identified from the OPLS-DA (VIP > 1) and differential metabolites identified from the univariate analyses (*p* < 0.05). The red stars or the red upward arrows represent significantly increased metabolites, while the blue stars or the blue downward arrows denote significantly decreased metabolites in the MODEL groups relative to the CON groups.

## Data Availability

Data is contained within the article or Supplementary Material.

## Supplementary Materials

The following supporting information can be downloaded at: https://www.mdpi.com/article/10.3390/molecules27186115/s1, Figure S1. Typical 1D ^1^H-NMR spectra of mouse cardiac tissue (850 MHz, 298 K, pH 7.4). Figure S2. 2D ^1^H-13C-HSQC spectrum of mouse cardiac tissue (850 MHz, 298 K, pH 7.4). Figure S3. OPLS-DA analyses of cardiac tissues for identifying significant metabolites primarily responsible for distinguishing metabolic profiles between pathologic groups and normal groups. Figure S4. Cross validation plots of the OPLS-DA models of the three pathological stages. Figure S5. Multivariate ROC analysis of cardiac tissues for identifying potential biomarkers in the three pathological stages compared to normal controls. Figure S6. Metabolic pathway analyses of the three pathological stages based on metabolite levels in cardiac tissues. Table S1. Resonance assignments of metabolites in 1D ^1^H-NMR spectra of mouse cardiac tissues. Table S2. Comparison of significant metabolites between cardiac tissues and sera derived from the six groups of mice.

## Sample Availability

Samples of the compounds are available from the authors.

## Abbreviations

The following abbreviations are used in this manuscript:

VMC	Viral myocarditis
DCM	Dilated cardiomyopathy
CVB3	Coxsackievirus B3
aVMC	Acute viral myocarditis
cVMC	Chronic viral myocarditis
CON	Normal controls
HF	Heart failure
PBS	Phosphate buffered saline
HCA	Hierarchical clustering analysis
PCA	Principal component analysis
RPTs	Response permutation tests
ROC	Receiver operating characteristic
PIV	Pathway impact values
LVEF	Left ventricle ejection fraction
BCAAs	Branch chain amino acids
PLS-DA	Partial least squares discriminant analysis
OPLS-DA	Orthogonal signal correction partial least-squares discriminant analysis
