# Supplementary material for: NMR-Based Metabolomic Analysis of Cardiac Tissues Clarifies Molecular Mechanisms of CVB3-Induced Viral Myocarditis and Dilated Cardiomyopathy
Source: Molecules. 2022 Sep 19;27(18):6115. doi: 10.3390/molecules27186115 (PMC9500976; doi:10.3390/molecules27186115)
Supplement: Supplementary file 1 [file molecules-27-06115-s001.zip › molecules-1859836-supplementary.pdf]

Supplementary Materials

Figure S1

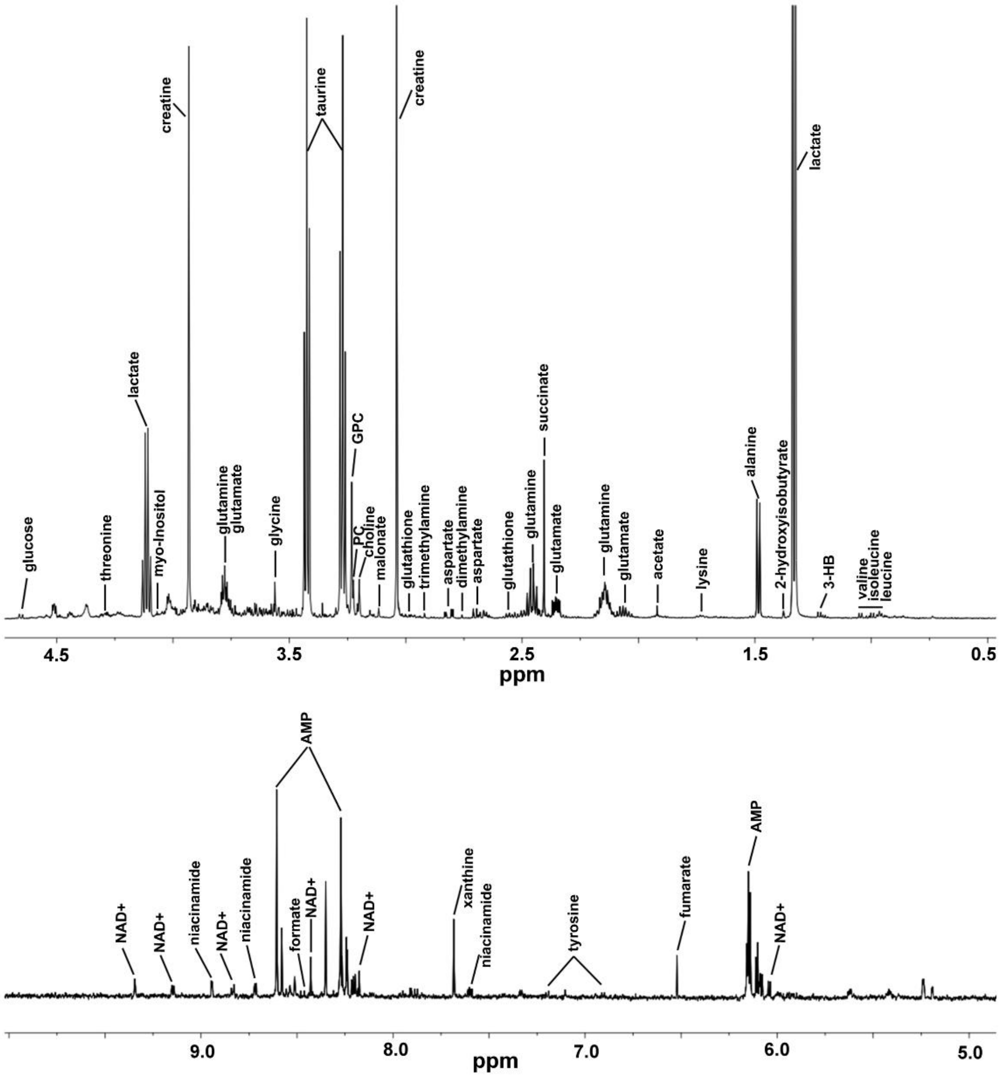

Figure S1. Typical 1D ^{1}H-NMR spectra of mouse cardiac tissue (850 MHz, 298 K, pH 7.4).

Figure S2

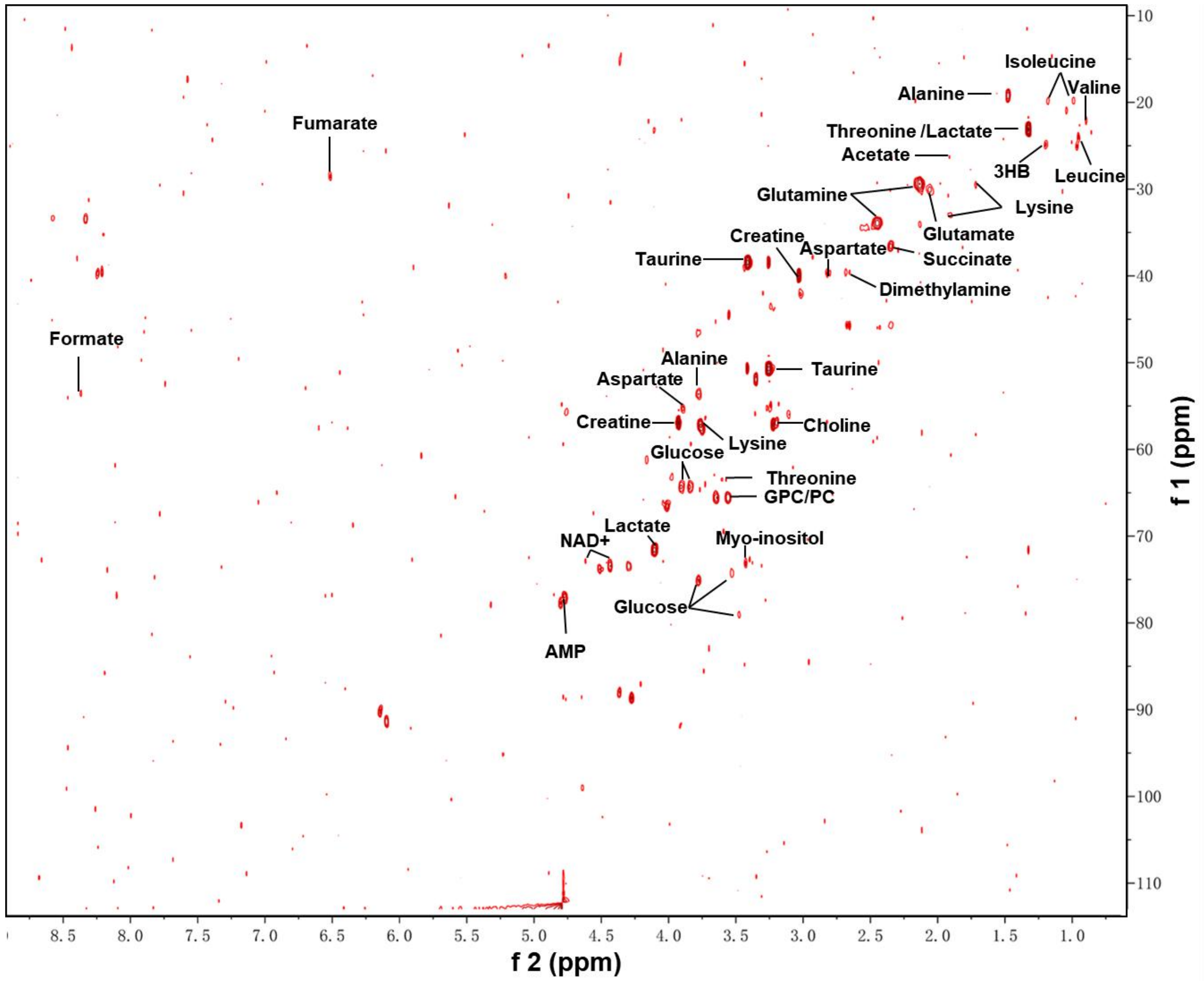

Figure S2. 2D ^{1}H-^{13}C-HSQC spectrum of mouse cardiac tissue (850 MHz, 298 K, pH 7.4). Corresponding abbreviations of metabolite names are shown in Table S1.

Figure S3

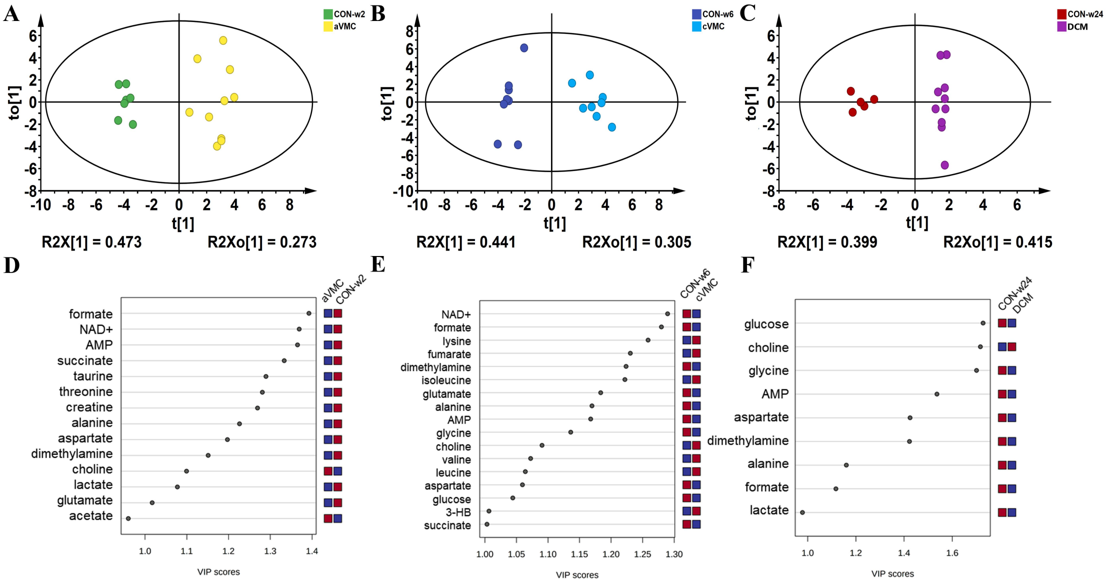

Figure S3. OPLS-DA analyses of caridac tissues for identifying significant metabolites primarily responsible for distinguishing metabolic profiles between pathologic groups and normal groups. (A, B, C) OPLS-DA scores plots of aVMC vs. CON-w2 (A), cVMC vs. CON-w6 (B), DCM vs. CON-w24 (C). (D, E, F) VIP scores of the OPLS-DA models of aVMC vs. CON-w2 (D), cVMC vs. CON-w6 (E), DCM vs. CON-w24 (F). The criterion of VIP >1 was used to identify significant metabolites. Red and blue squares represent increased and decreased metabolites, respectively.

Figure S4

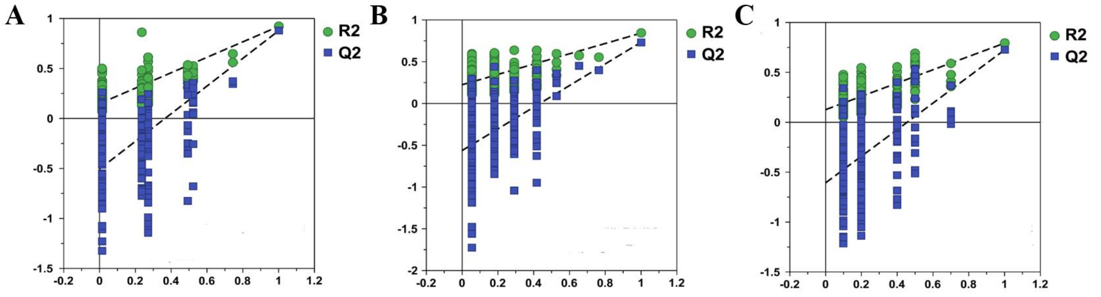

Figure S4. Cross validation plots of the OPLS-DA models of the three pathological stages. (A) aVMC vs. CON-w2; (B) cVMC vs. CON-w6; (C) DCM vs. CON-w24. Robustnesses of the OPLS-DA models were cross-validated by using random permutation tests (n=200). The green circle is R2, denoting the explained variance of the model. The blue square is Q2, standing for the predictive ability of the model. The obtained R2, Q2 and *p* values were 0.921, 0.878 and 0.005 for aVMC vs. CON-w2, 0.842, 0.733 and 0.005 for cVMC vs. CON-w6, 0.792, 0.724 and 0.005 for DCM vs. CON-W24.

Figure S5

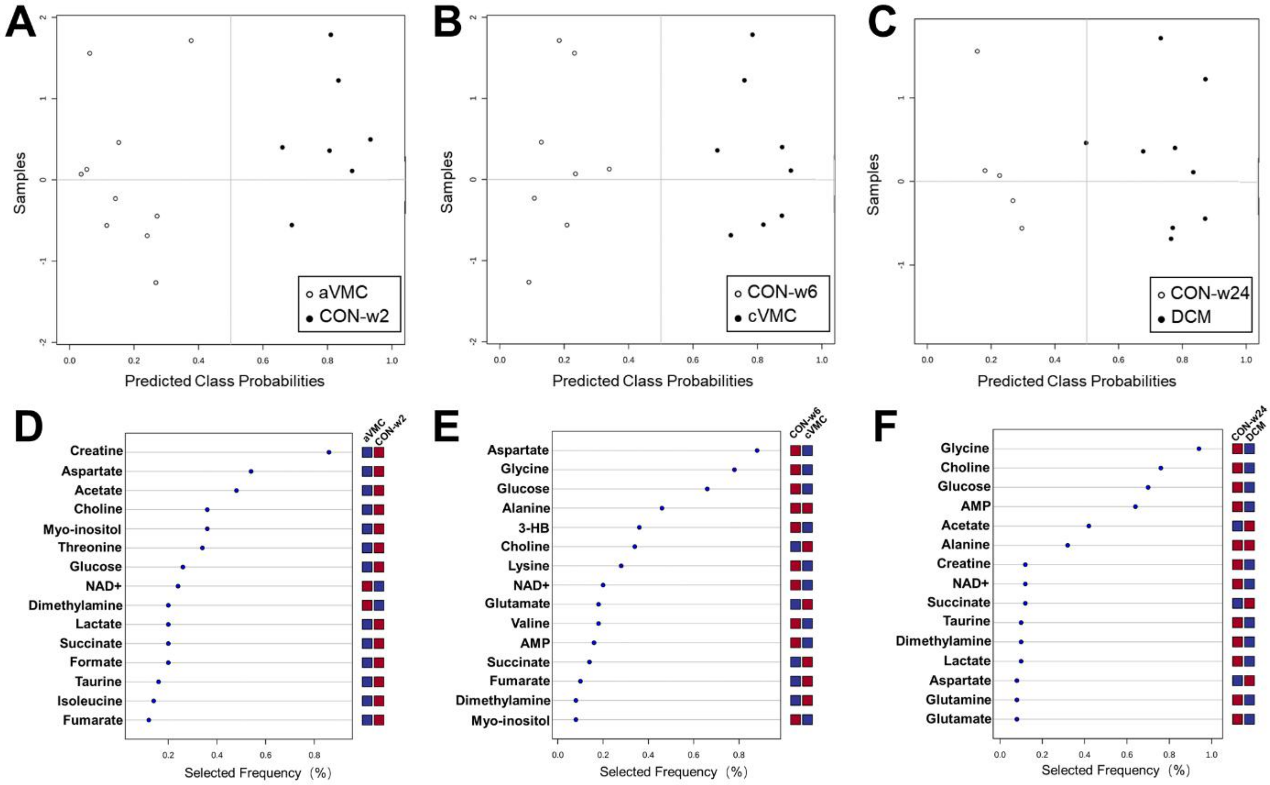

Figure S5. Multivariate ROC analysis of cardiac tissues for identifying potential biomarkers in the three pathological stages compared to normal controls. (A-C) Predicted class probabilities for each sample using the base classifier based on AUC of the ROC curve built with five metabolites. (A) aVMC vs. CON-w2; (B) cVMC vs. CON-w6; (C) DMC vs. CON-w24. (D-F) Metabolites ranked by the frequencies of being selected during cross validation. (D) aVMC vs. CON-w2; (E) cVMC vs. CON-w6; (F) DMC vs. CON-w24.

Figure S6

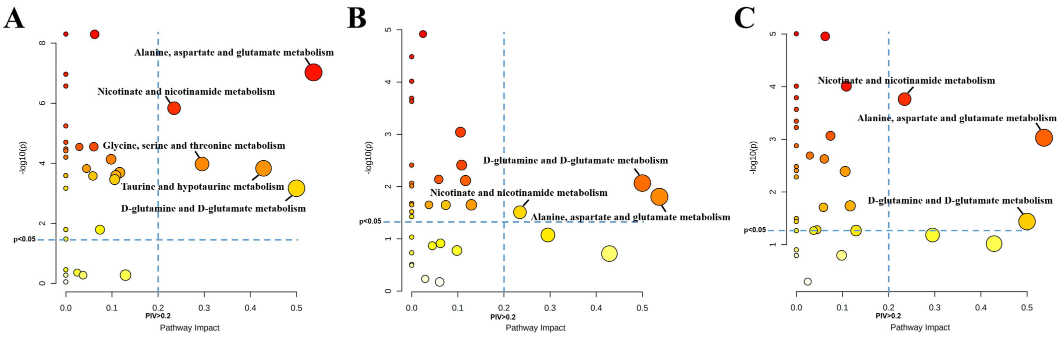

Figure S6. Metabolic pathway analyses of the three pathological stages based on metabolite levels in cardiac tissues. (A) aVMC vs. CON-w2; (B) cVMC vs. CON-w6; (C) DCM vs. CON-w24. Pathway impact values (PIVs) were calculated from pathway topology analysis, and *p* values were computed from metabolite set enrichment analysis. Two criteria were used to identify significantly altered metabolic pathways: pathway impact values > 0.2, *p* values < 0.05.

Table S1. Resonance assignments of metabolites in 1D ^{1}H-NMR spectra of mouse cardiac tissues.

Num.	Metabolite	δ ^{1}H (ppm) and multiplicity	Moieties
1	leucine	0.96(d), 0.97(d), 1.69(m), 1.70(m), 1.73(m), 3.73(m)	α-CH_3, α-CH_3, γ-CH, β-CH_2, α-CH
2	isoleucine	0.94(t), 1.01(d), 1.21(m), 1.42(m), 2.00(m), 3.67(d)	δ-CH_3, γ-CH_3, half γ-CH_2, half γ-CH_2, β-CH, α-CH
3	valine	0.99(d), 1.05(d), 2.26(m), 3.60(d)	γ-CH_3, γ-CH_3, β-CH, α-CH
4	3-HB	1.197(d), 2.314(m), 2,394(m), 4.142(m)	γ-CH_3, β-CH_2, γ-CH
5	lactate	1.33(d), 4.11(q)	β-CH_3, α-CH
6	threonine	1.30(d), 3.58(d), 4.24(m)	γ-CH_2, β-CH
7	alanine	1.47(d), 3.78(q)	β-CH_3, α-CH
8	lysine	1.43(m), 1.49(m), 1.70(m), 1.91(m), 3.02(t), 3.75(t)	half γ-CH_2, half γ-CH_2, δ-CH_2, β-CH_2, ε-CH_2, α-CH
9	acetate	1.91(s)	CH_3
10	succinate	2.41(s)	CH
11	glutamine	2.13(m), 2.45(m), 3.77(t)	γ-CH_2, β-CH_2, α-CH
12	glutamate	2.08(m),2.12(m),2.34(m),2.37(m),3.75 (m)	Half β-$CH_{2,}$ Half β-$CH_{2,}$ Half γ-CH_2, Half γ-CH_2,α-CH
13	aspartate	2.68(dd),2.81(dd),3.90(dd)	β-$CH_{2,}$ α-CH
14	creatine	3.04(s), 3.93(s)	N-CH_3, α-CH_2
15	GPC/PC	3.23(s), 3.60(dd), 3.68(dd), 3.87(m), 3.94(m), 4.33(m)	N-$(CH_3)_3$, half $^1CH_2$, $^2CH_2$, half $^1CH_2$, half $^3CH_2$, half $^3CH_2$, $^1CH_2$
16	taurine	3.24(t), 3.41(t)	$^1CH_2$, $^2CH_2$
17	glycine	3.57(s)	α-CH_2
18	dimethylamine	2.50(s)	CH_3
19	glucose	5.22(d), 4.63(d), 3.9(dd), 3.72(m), 3.48(m), 3.22(dd)	β(H_2, H_3, H_5),

			α (H_2, H_3, H_6)
20	Myo-inositol	3.28(t),3.53(dd),3.63(t),4.07(t)	^{2}CH,^{4,6}CH,^{1,3}CH,^{5}CH
21	NAD^+	6.03(d),6.08(s),8.16(s),8.20(m),8.41(s), 8.22(d),9.13(d),9.32(s)	NH_2,NH_2(CO),d-CH,β-CH, γ-CH, α-CH
22	choline	3.21(s), 3.51(dd), 4.04(t)	N-$(CH_3)_3$, α-CH_2, CH_2OH
23	fumarate	6.51(s)	CH
24	AMP	6.14(d),8.27(s),8.58(s)	NH_2, δ-CH,^{2}CH
25	formate	8.46（s）	CH

Table S2. Comparison of significant metabolites between cardiac tissues and sera derived from the six groups of mice.

	aVMC vs.CON-w2		cVMC vs. CON-w6		DCM vs.CON-w24	
	sera	heart	sera	heart	sera	heart
**Amino acid metabolism**						
leucine	--	--	↑	↑↑	↓↓	↓
isoleucine	--	--	↑	↑↑↑	--	--
valine	↓↓↓	--	↑↑	↑↑	--	--
glycine	↓↓	↓	--	↓	↑↑	↓↓↓
lysine	--	--	↑	↑↑↑	--	--
taurine	↓↓↓	↓↓↓	↑	--	↑↑↑	--
**Carbohydrate metabolism**						
glucose	↓	--	↓	↓	↑↑↑	↓↓↓
lactate	--	↓↓↓	--	--	↑	↓
succinate	--	↓↓↓	--	--	↑	↓↓↓
**Choline phosphorylation metabolism**						
GPC	↓	--	--	--	↓	↓↓

Note: Arrows ↓/↑ mean that the changes of relative metabolite levels in the Model groups are highly significant (↓↓↓/↑↑↑, p<0.001), very significant (↓↓/↑↑, p<0.01), significant (↓/↑, p<0.05) relative to their normal controls (aVMC *vs.* CON-w2, cVMC *vs.* CON-w6, DCM *vs.* CON-w24). The red upward arrow ↑ and blue downward arrow ↓ denote significantly increased and decreased metabolites, respectively. Arrows in the yellow background and the green background represent the consistent and opposite changing trends between cardiac tissues and sera, respectively.
